# A novel miRNA-disease association prediction model using dual random walk with restart and space projection federated method

**DOI:** 10.1371/journal.pone.0252971

**Published:** 2021-06-17

**Authors:** Ang Li, Yingwei Deng, Yan Tan, Min Chen

**Affiliations:** 1 Hunan Institute of Technology, School of Computer Science and Technology, Hengyang, China; 2 Hainan Key Laboratory for Computational Science and Application, Haikou, China; Chinese Academy of Sciences, CHINA

## Abstract

A large number of studies have shown that the variation and disorder of miRNAs are important causes of diseases. The recognition of disease-related miRNAs has become an important topic in the field of biological research. However, the identification of disease-related miRNAs by biological experiments is expensive and time consuming. Thus, computational prediction models that predict disease-related miRNAs must be developed. A novel network projection-based dual random walk with restart (NPRWR) was used to predict potential disease-related miRNAs. The NPRWR model aims to estimate and accurately predict miRNA–disease associations by using dual random walk with restart and network projection technology, respectively. The leave-one-out cross validation (LOOCV) was adopted to evaluate the prediction performance of NPRWR. The results show that the area under the receiver operating characteristic curve(AUC) of NPRWR was 0.9029, which is superior to that of other advanced miRNA–disease associated prediction methods. In addition, lung and kidney neoplasms were selected to present a case study. Among the first 50 miRNAs predicted, 50 and 49 miRNAs have been proven by in databases or relevant literature. Moreover, NPRWR can be used to predict isolated diseases and new miRNAs. LOOCV and the case study achieved good prediction results. Thus, NPRWR will become an effective and accurate disease–miRNA association prediction model.

## 1. Introduction

MiRNAs are a kind of single-stranded, non-coding RNA with a length of about 20–25 nucleotides. miRNAs combine with 3′untranslated regions and inhibit the translation of target mRNAs, showing a significant influence on the expression of genes after transcription [1–3]. miRNAs are also involved in the physiological and pathological processes of mammals [4]; the development, differentiation, growth, and metabolism of cells are closely related to miRNAs [5]. In addition, studies have shown that miRNAs play an important role in the pathogenesis of human diseases. The transfection of miRNA-101 can affect the induction and expression of ubiquitin ligase HECTH9 in acute myeloid leukemia cells [6]; miRNA-21, an exosome derived from hepatocellular carcinoma, promotes tumor progression by transforming hepatic stellate cells into cancer-associated fibroblasts [7]. Therefore, revealing the potential relationship between miRNAs and human diseases can help in the diagnosis, treatment, prognosis, and prevention of diseases. However, determining the association between miRNAs and diseases by biological experiments is time-consuming and laborious. Therefore, computational models should be used to predict potential miRNA–disease associations to offer guidance in biological experiments, thus saving cost and time. As a result, our understanding of life processes at the RNA level can be accelerated.

With the constant accumulation of miRNA, disease, and miRNA–disease association data, numerous computational methods have emerged and been used to predict miRNA–disease associations. Jiang et al. [8] computed the functional similarity of miRNAs by using miRNA target genes and ranked disease-associated miRNAs through hypergeometric distribution. Li et al. [9] predicted miRNA–disease associations by using the information on the miRNA and disease targets. Xu et al. [10] ranked disease-associated miRNAs on a miRNA-target dysregulated network by using support vector machine (SVM). Shi et al. [11] predicted miRNA–disease associations on a protein–protein interaction network by using the information on miRNA target genes. These methods have attained certain prediction results. However, all the above methods use target gene information. Therefore, a high false-positive defect is possible with their use.

Based on the hypothesis that functionally similar miRNAs are often associated with similar diseases, and vice versa, several scholars successfully implemented random walk with restart on their own heterogeneous networks to predict potential miRNA–disease associations [12–14]. Chen et al. [15] predicted miRNA–disease associations by using random walk with restart. This procedure is a globally applied method. Afterward, numerous improved random walk algorithms have been used in the prediction of miRNA–disease associations. Xuan et al. [16] proposed an improved random walk model(MIDP). MIDP can predict new diseases without any association information.

Most scholars predict miRNA–disease associations by using the graph theory [17]. You et al. [18] used depth-first search algorithm on a miRNA–disease heterogeneous graph to acquire path information for the prediction of potential miRNA–disease associations. Chen et al. [19] predicted miRNA–disease associations through calculating within-scores and between-scores of miRNA–disease groups. Chen et al. [20] identified miRNA–disease associations through acquiring iteration information on a heterogeneous graph. Chen et al. [21] predicted miRNA–disease associations by using Jaccard similarity and hubness-aware regression on a bipartite graph; Chen et al. [22] predicted miRNA–disease associations by using common neighbor information from a bipartite graph. Chen et al. [23] and Zhang et al. [24,25] predicted miRNA–disease associations by using network projection on a bipartite graph. Chen et al. [26] and Li et al. [27] predicted miRNA–disease associations by using label propagation algorithm in heterogeneous networks. Li et al. [28] predicted miRNA–disease associations by using DeepWalk on heterogeneous networks. Zhang et al. [29] constructed a multiple meta-path fusion graph embedding model through integrating nodes and edge information to predict miRNA–disease associations. Lv et al. [30] predicted disease-associated miRNAs through solving a meta-path in a heterogeneous network composed of miRNA similarity, diseases similarity, and miRNA–disease associations. However, this method failed to solve the problems on parameter selection. If the machine learning method is used to solve the optimal parameters, then the prediction performance will be improved.

Numerous scholars have used machine learning to predict miRNA–disease associations. Zou et al. [31] deduced potential miRNA–disease associations by introducing two prediction models, namely, KATZ and CATAPULT. Chen et al. [32] first proposed a prediction model for miRNA–disease association, that is, EGBMMDA, based on a decision tree model. Then, Chen et al. [33] proposed a new prediction model for miRNA–disease associations, called EDTMDA, based on a decision tree ensemble. Zhao et al. [34] proposed an adaptive enhanced miRNA–disease association prediction model. This method is used to first cluster unknown samples by k-means clustering to obtain negative samples and then predict associations by using a decision tree. Chen et al. [35] predicted disease-associated miRNAs by means of random forest. Chen et al. [36] put forward a computational method based on K-nearest neighbor (KNN), that is, RKNNMDA. In this method, a support vector mechanism is used in re-ranking to acquire the prediction scores. Thereafter, Wang et al. [37] designed an efficient negative-sample extraction strategy and used a SVM to make predictions. Wu et al. [38] constructed a hypergraph using KNN and K-means algorithm to make predictions. However, the prediction accuracy of this method for new miRNAs is low.

The issue on miRNA–disease association can be regarded as a binary classification problem given the lack of negative samples. On this basis, scholars have proposed various semi-supervised machine learning methods. Chen et al. [39] proposed a prediction model based on regularized least squares algorithm: RLSMDA. RLSMDA can be used for prediction without using any negative sample. However, this model is highly dependent on parameters. For further improvement, Chen et al. [40] proposed a graph regression prediction model based on singular value decomposition and partial least squares regression. Chen et al. [41] predicted miRNA–disease associations based on Laplacian regularized sparse subspace learning. Luo et al. [42] proposed KRLSM, a miRNA–disease association prediction model based on Kronecker regularized least squares. However, this model highly depends on weight coefficients of different similarity measures. Li et al. [43] predicted miRNA–disease associations by using Kronecker kernel matrix dimension reduction. Pasquier and Gardes performed dimension reduction for multiple miRNA-related association networks by using singular value decomposition and predicted miRNA–disease associations through calculating cosine similarity.

Against the insufficiency of miRNA similarity data, the rare relationship between known miRNA and diseases, and almost zero negative sample [44], Zeng et al. [45] proposed a miRNA–disease association prediction method based on a matrix completion algorithm. This method provides a new idea to solve the problem of insufficient miRNA–disease association data, and it can be used in the prediction of novel diseases and pathogenic miRNA. Chen et al. [46] treated redundant information for miRNA–disease neighbor matrix by using matrix factorization to predict disease-associated miRNAs. Li et al. [47] proposed MCMDA, a miRNA–disease association prediction model based on matrix completion. This model requires no negative association. Chen et al. [48] proposed IMCMDA, an inductive matrix completion integrating miRNA functional similarity and semantic similarity of diseases. Xuan et al. [49] proposed two kinds of non-negative matrix factorizations to predict disease-associated miRNAs. Zhao et al. [50] developed the associated prediction model SNMFMDA by combining Kronecker regularized least square with symmetric non-negative matrix factorization.

Xiao et al. [51] proposed GRNMF, a miRNA-disease association prediction algorithm based on graph regularized nonnegative matrix factorization. However, the prediction result of this method highly depends on selected parameters. Xu et al. [52] designed PMFMDA, a prediction method based on the probability matrix factorization prediction method. PMFMDA can integrate the similarity of miRNAs and diseases and construct a probability matrix factorization algorithm by using known an association matrix and integrating a similarity matrix to deduce new miRNA–disease associations. Wang et al. [53] integrated the neural network matrix factorization and multi-layer perception into the deep collaborative filtering framework to predict miRNA–disease associations. However, this method shows no enhancement in dealing with the problem on negative sample selection.

Scholars have applied deep learning to the prediction of miRNA–disease association. Xuan et al. [54] first proposed a method based on double convolution neural network (CNNDMP) to predict miRNA–disease associations. Then, they put forward a prediction method based on network representation learning and convolutional neural network (CNNMDA) [55]. Ding et al. [56] developed a deep learning model based on variational graph auto-encoder. However, this model covers two deep learning networks. Thus, the complexity of the algorithm is high.

Chen et al. [57] predicted miRNA–disease associations with RBMMMDA method by using a restricted Mansman machine. Compared with previous methods, RBMMMDA can not only predict miRNA–disease associations but also acquire the type of association. However, RBMMMDA only uses known miRNA–disease association information, which prevents it from achieving an excellent performance. Zhang et al. [58] predicted the information type of miRNA–disease associations by using label propagation. However, the correlation between association types is ignored with this method. Huang et al. [59] expressed miRNA-disease-type triplets as a tensor and solved the prediction task by using the tensor decomposition method. However, this method remains limited by defects with few known associations, resulting in a low prediction accuracy.

In conclusion, although various prediction methods for miRNA–disease associations have emerged, several limitations still exist. First, most methods cannot predict isolated diseases and novel miRNAs. Second, a number of methods require negative samples for miRNA–disease associations, but negative sample selection presents difficulty.

In addition, several lncRNA–disease association prediction methods [60–64], drug–disease association prediction method [65], and several related computational methods [66–69] can provide help in the prediction of miRNA–disease associations. In this paper, a new method, network projection-based dual random walk with restart (NPRWR), which integrates dual random walk with restart and network projection technology, is proposed to predict potential miRNA–disease associations. First, NPRWR was used to acquire the miRNA–disease association prediction matrix based on dual random walk with restart to compensate for the lack of known miRNA–disease association data. Then, the network projection method was implemented to acquire the final association prediction matrix. The experimental results show that NPRWR has a better prediction effect compared with other algorithms with excellent performance.

## 2. Materials and methods

### 2.1. Method overview

**Fig 1:**
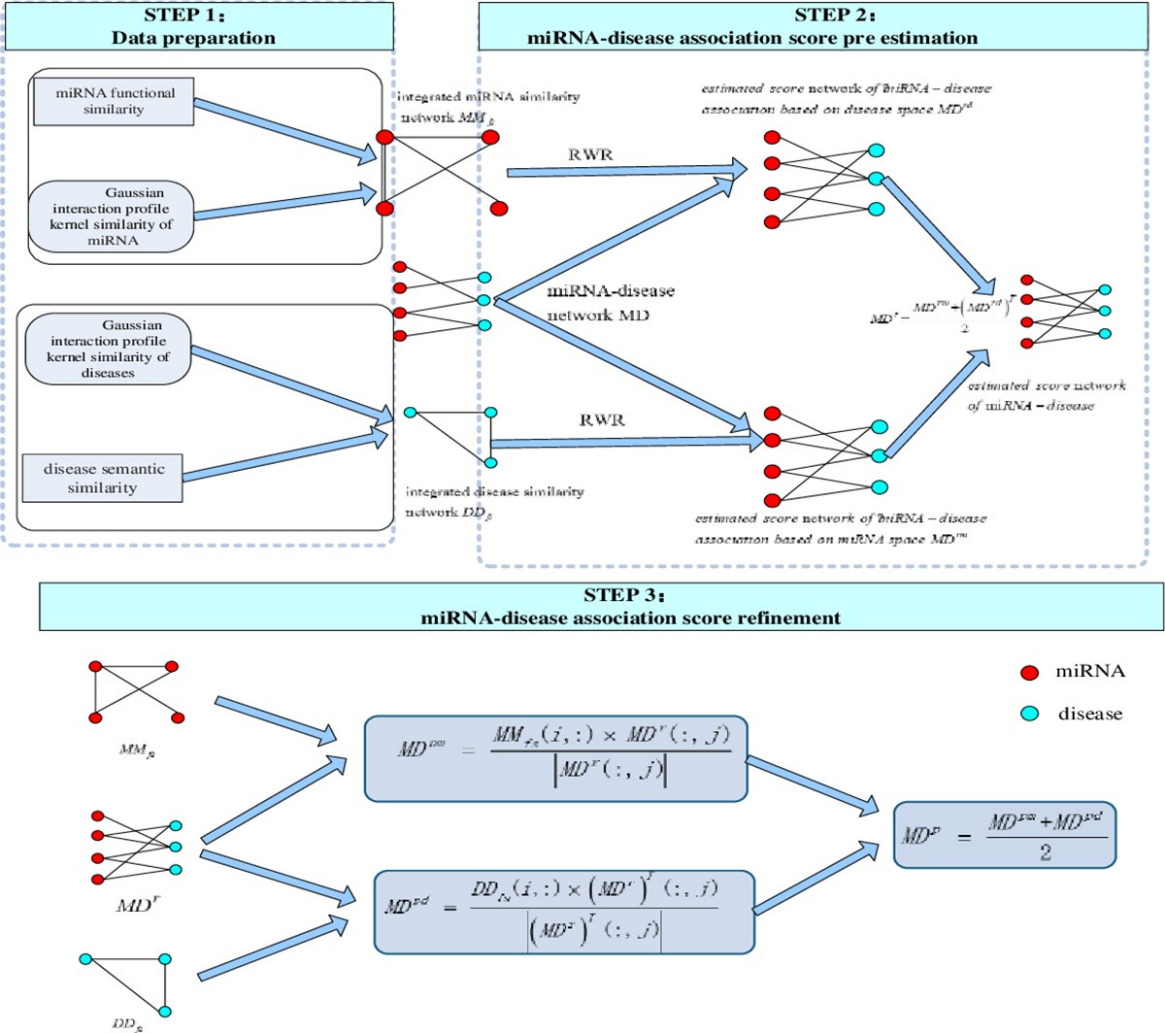
Flowchart of the whole modeling procedure.

NPRWR mainly includes three steps. Fig 1 shows the algorithm flow chart. (1) Data preparation. Disease similarity integrated is constructed by using disease semantic similarity and Gaussian interaction profile kernel similarity of diseases, and integrated miRNA similarity is constructed by using miRNA functional similarity and Gaussian interaction profile kernel similarity of miRNA. (2) miRNA–disease association prediction. Dual random walk with restart is implemented in the integrated miRNA network and integrated disease network, and two stable distribution vectors are obtained. Then, the two distribution vectors are integrated to obtain the miRNA–disease association prediction score. (3) Refined prediction. The miRNA–disease association prediction scores are projected in miRNA and disease spaces, and the two projection scores are integrated as the final miRNA–disease association prediction score.

### 2.2. Data source

#### 2.2.1. MiRNA–disease association

To study the association between miRNA and human diseases, Li et al. [70] established a HMDD database to record miRNA–human disease associations. The associations between 383 human diseases and 495 miRNAs were extracted from this database. A total of 5430 miRNA–disease associations were confirmed experimentally, as represented by matrix ${MD}^{n_{m}\times n_{d}}$. If an association was verified experimentally between the miRNA node d_j_,MD(i,j) and disease node d_j_,MD(i,j), the value was set to 1; otherwise, the value was set to 0.

#### 2.2.2. Disease semantic similarity

Wang et al. [71] proposed a disease semantic similarity measurement method based on the disease classification information described by MeSH. Each disease is described as a directed acyclic graph (DAG) with the hierarchical structure in MeSH. According to the DAGs of two diseases described by MeSH, the semantic similarity between the diseases can be measured. This method is used to express the semantic similarity between two diseases, as represented by matrix ${DD}^{n_{d}\times n_{d}}$.

#### 2.2.3. MiRNA functional similarity

Based on the hypothesis that miRNAs with similar functions are associated with diseases with similar phenotypes, and vice versa, Wang et al. [71] proposed a method to calculate the functional similarity between miRNAs. This method was successfully applied to the prediction of disease-associated miRNAs. Thus, this method was adopted to calculate the functional similarity between miRNAs, and matrix ${MM}^{n_{m}\times n_{m}}$ was used to represent the functional similarity between miRNAs.

#### 2.2.4. Gaussian interaction profile kernel similarity of diseases

When disease semantic similarity is adopted to measure the similarity between diseases, given the missing data, the semantic similarity between various diseases is 0. The concept of Gaussian interaction profile kernel similarity between diseases is introduced to solve this problem.

$$GD(i,j)=exp({-\gamma }_{d}{\left\|MD(:,i)-MD(:,j)\right\|}^{2})$$ (1)

where *GD*(*i*,*j*) refers to the Gaussian interaction profile kernel similarity between diseases *d*_*i*_ and *d*_*j*_; *MD*(:,*i*) refers to column i of matrix ${MD}^{n_{m}\times n_{d}}$; parameter *γ*_1_ is used to control the kernel bandwidth of Gaussian interaction profile kernel similarity, and it can be calculated by the using Formula (2):

$$\gamma_{d}=\frac{\gamma_{d}^{\prime }}{\frac{1}{n_{d}}\sum_{i=1}^{n_{d}}{\left\|MD(:,i)\right\|}^{2}}$$ (2)

where $\gamma_{d}^{\prime }$ is set to 1.

Similarly, the Gaussian interaction profile kernel similarity between miRNAs is calculated as below:

$$GM(i,j)=exp({-\gamma }_{1}{\left\|MD(i,:)-MD(j,:)\right\|}^{2})$$ (3)

where *GM*(*i*,*j*) refers to Gaussian interaction profile kernel similarity between miRNAs *m*_*i*_ and *m*_*j*_; *MD*(*i*,:) refers to row *i* of matrix ${MD}^{n_{m}\times n_{d}}$; parameter *γ*_1_ is used to control the kernel bandwidth of Gaussian interaction profile kernel similarity, and it can be calculated by using Formula (4):

$$\gamma_{1}=\frac{\gamma_{1}^{\prime }}{\frac{1}{n_{m}}\sum_{i=1}^{n_{m}}{\left\|MD(i,:)\right\|}^{2}}$$ (4)

where $\gamma_{1}^{\prime }$ is set to 1.

#### 2.2.5. Disease (miRNA) integrated similarity

Finally, the disease similarity is obtained through integrating disease semantic similarity with disease Gaussian interaction profile kernel similarity, and miRNA similarity is obtained through integrating the functional similarity of miRNA with miRNA Gaussian interaction profile kernel similarity. The formula is as below:

$${DD}_{fs}(i,j)=\left\{ \begin{array}{l} DD(i,j),\;DD(i,j)\neq 0\\ GD(i,j),\;DD(i,j)=0 \end{array}\right.$$ (5)

$${MM}_{fs}(i,j)=\left\{ \begin{array}{l} MM(i,j),\;MM(i,j)\neq 0\\ GM(i,j),\;MM(i,j)=0 \end{array}\right.$$ (6)

### 2.3. miRNA–disease association pre estimation

To solve the sparsity problem of a known miRNA–disease association network, we first walked in the miRNA similarity network by using random walk with restart and then captured the stable information distribution to represent the association degree between the miRNA and disease nodes. The formula is as below:

$${\left({MD}^{rm}(:,j)\right)}_{t+1}=(1-\gamma )\times \bar{{MM}_{fs}}\times {\left({MD}^{rm}(:,j)\right)}_{t}+\gamma \times \bar{MD(:,j)}$$ (7)

where $\bar{MD(:,j)}$ refers to the information in column *j* after matrix *MD* is normalized in the column. The vector in this column refers to the seed sequence of the association between disease *d*_*j*_ in the disease node and all miRNA nodes; $\bar{{MM}_{fs}}$ refers to the column normalization matrix of *MM*_*fs*_ integrating miRNA functional similarity; *γ* refers to restart probability; (*MD*^*rm*^(:,*j*))_*t*_ vector refers to the information distribution after t times of iteration. After several iterations, if the probability space reaches the stable state, ${\left({MD}^{rm}(:,j)\right)}_{\propto }(|{\left({MD}^{rm}(:,j)\right)}_{t+1}-{\left({MD}^{rm}(:,j)\right)}_{t}|<10^{-6})$, then the iteration is stopped. In the stable state, the values of this vector refer to the scores of associations between disease *d*_*j*_ and all miRNAs. The pre-estimated score of miRNA–disease association by random walk algorithm based on miRNA similarity network is represented by matrix *MD*^*rm*^.

Similarly, the random walk with restart was adopted to walk in the disease similarity network, and the association pre-estimated value by random walk with restart based on disease network was obtained. The formula is as below:

$${\left({MD}^{rd}(:,i)\right)}_{t+1}=(1-ŋ)\times \bar{{DD}_{fs}}\times {\left({MD}^{rd}(:,i)\right)}_{t}+ŋ\times \bar{{MD}^{T}(:i)}$$ (8)

where *MD*^*T*^ refers to the transpose matrix of *MD*; $\bar{{MD}^{T}(:i)}$ refers to the information in column *i* after matrix *MD*^*T*^ is normalized in the column. This vector denotes the seed sequence of the association between miRNA node *m*_*i*_ and all disease nodes; $\bar{{DD}_{fs}}$ corresponds to the column normalization matrix of *DD*_*fs*_ integrating miRNA functional similarity; ŋ indicates restart probability; (*MD*^*rd*^(:,*i*))_*t*+1_ vector represents the information distribution after *t* times of iteration. After several iterations, if the probability space reaches the stable state, ${\left({MD}^{rd}(:,i)\right)}_{\propto }(|{\left({MD}^{rm}(:,i)\right)}_{t+1}-{\left({MD}^{rm}(:,i)\right)}_{t}|<10^{-0})$, then the iteration is stopped. The values of this vector in the stable state are the scores of associations between miRNA node *m*_*i*_ and all disease nodes. The pre-estimated score of miRNA-disease association by random walk algorithm based on disease similarity network is represented by *MD*^*rd*^.

Then, the miRNA-disease prediction score based on random walk algorithm was obtained by integrating the prediction score by miRNA network-based random walk algorithm and the prediction score by disease network-based random walk algorithm.


$${MD}^{r}=\frac{{MD}^{rm}+{\left({MD}^{rd}\right)}^{T}}{2}$$ (9)

### 2.4. Refined prediction of miRNA–disease association

Given that the random walk algorithm was adopted to obtain miRNA–disease prediction score, the network projection was used to obtain the final prediction score.

First, the miRNA similarity network was used to project on the miRNA–disease prediction score network, and the projection score based on the miRNA similarity network was obtained:

$${MD}^{pm}=\frac{{MM}_{fs}(i,:)\times {MD}^{r}(:,j)}{\left|{MD}^{r}(:,j)\right|}$$ (10)

Then, disease similarity network was used to project on the miRNA–disease prediction score network, and the projection score based on the disease similarity network was obtained:

$${MD}^{pd}=\frac{{DD}_{fs}(i,:)\times {\left({MD}^{r}\right)}^{T}(:,j)}{\left|{\left({MD}^{r}\right)}^{T}(:,j)\right|}$$ (11)

Finally, the final prediction score was obtained through integrating the projection score based on miRNA similarity network and the projection score based on disease similarity network:

$${MD}^{p}=\frac{{MD}^{pm}+{MD}^{pd}}{2}$$ (12)

## 3. Results

### 3.1. Evaluation method

LOOCV was adopted to evaluate the performance of NPRWR. Specifically, each pair of miRNA–disease association was used as a test sample, and the remaining associations were used as training samples for model training. Each pair of miRNA–disease association was tested once as a test sample. The receiver operating characteristic (ROC) curve and AUC values were used to evaluate the performance indicators of the prediction model. The ROC curve, also called the working characteristic curve or sensitivity curve of the subjects, is a comprehensive index reflecting sensitivity and specificity. If the ROC curve is convex and close to the upper left corner, the AUC value is large, and an excellent prediction performance is obtained.

### 3.2. Parameter selection

In this section, we mainly aim to discuss the effect of restart parameters γ and ŋ on the prediction performance of NPRWR. In this paper, for simplicity, two restart parameters were set to have the same size. To show the effect of parameters on the prediction performance of NPRWR, we increased the restart parameter from 0.1 to 0.9 with the step length of 0.1 to calculate its AUC value.

**Fig 2:**
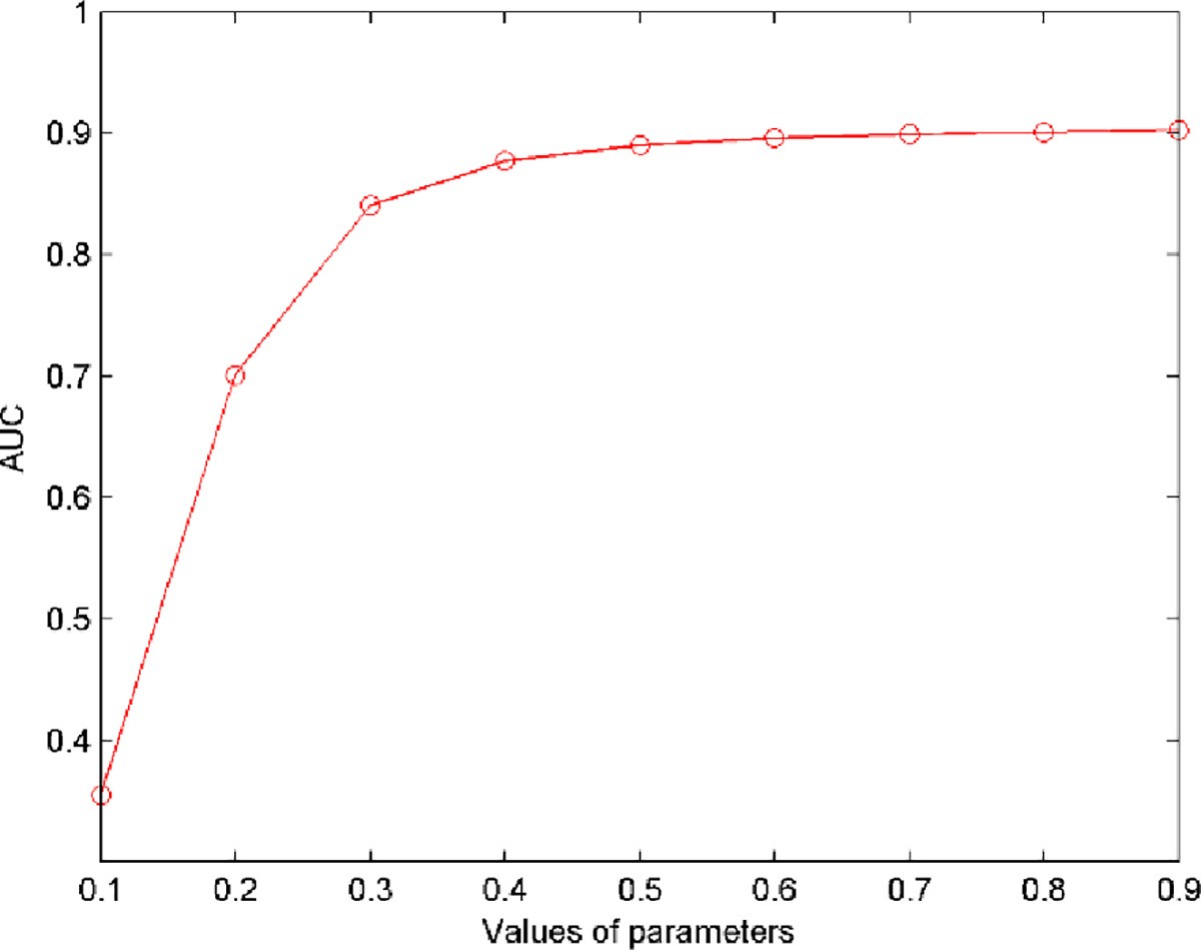
Influence of parameter variations on prediction accuracy.

Fig 2 describes the changes in the AUC value of NPRWR under different parameter values. The figure also shows that when the restart parameter increased from 0.1 to 0.9, the AUC value increased from 0.3548 to 0.9029. Therefore, 0.9 was considered the final value of the parameter.

### 3.3. Comparison with other prediction models

**Fig 3:**
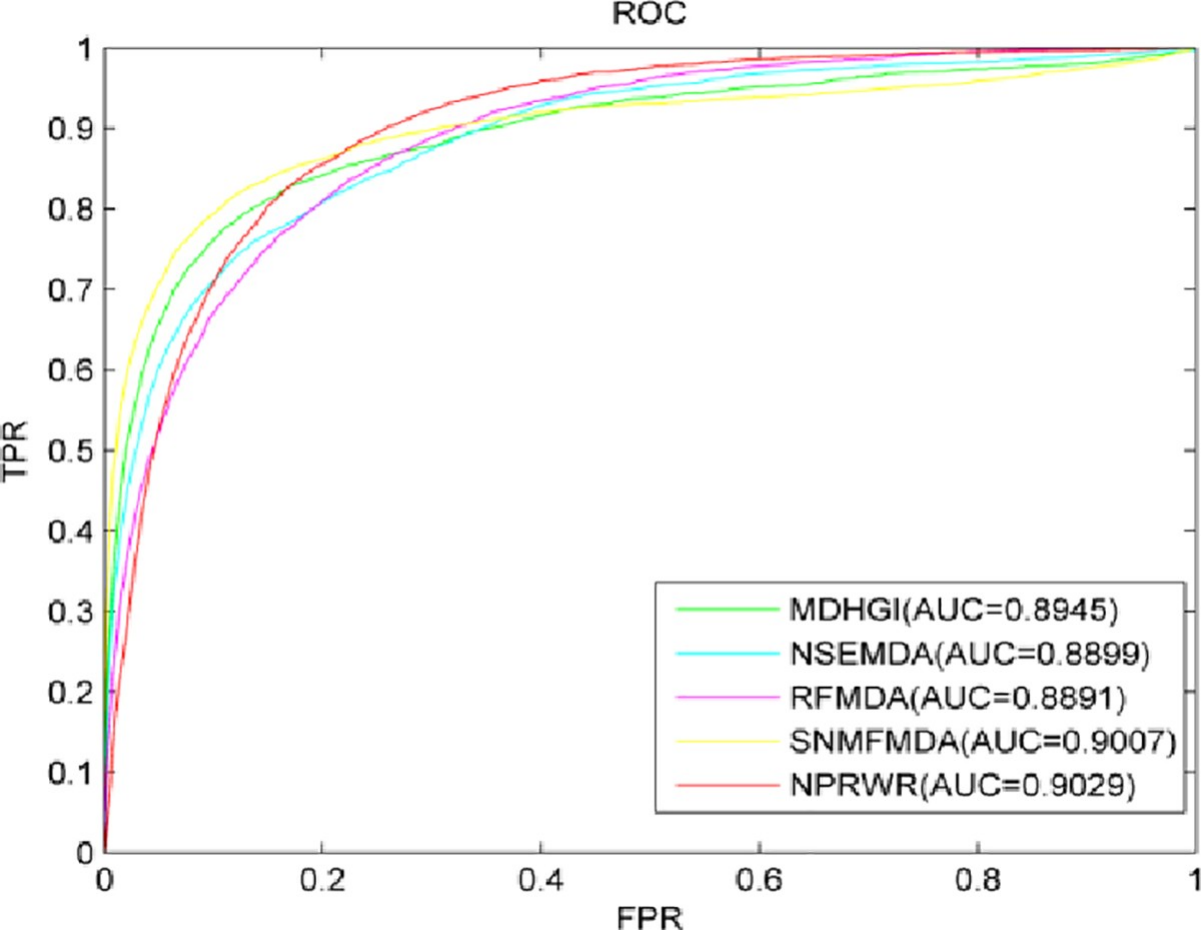
ROC curves and AUC values of NPRWR and other five methods.

MDHGI [46], NSEMDA [37], RFMDA [35], and SNMFMDA [50] are disease–miRNA prediction models with excellent performance. MDHGI makes prediction by using matrix decomposition and heterogeneous graph inference; NSEMDA proposes a novel negative-sample extraction strategy and makes predictions by using SVM. The RFMDA makes predictions by using random forest; SNMFMDA first fill the similarity matrix symmetrically during negative matrix factorization and then solves the association probability by using Kronecker product regularized least square method to make predictions. These methods, similar to NPRWR, aim to combine the miRNA functional similarity, disease semantic similarity, and Gaussian interaction profile kernel similarity for diseases and miRNAs by using known miRNA–disease association information to make predictions. A comparative experiment was carried out in this study. Against NPRWR, MDHGI, NSEMDA, RFMDA, and SNMFMDA methods, LOOCV was deployed on the data set to evaluate their prediction performance. The optimal parameters of MDHGI, NSEMDA, RFMDA, and SNMFMDA were set in accordance with the description of authors in relevant literature. Fig 3 shows the ROC curves and AUC values in LOOCV by these methods. The AUC value of NPRWR was 0.9029, whereas those of MDHGI, NSEMDA, RFMDA, and SNMFMDA were 0.8945, 0.8899, 0.8891, and 0.9007, respectively. The comparison showed that NPRWR achieved the best prediction effect. Moreover, compared with MDHGI, NSEMDA, RFMDA, and SNMFMDA, NPRWR is simple and does not require negative samples. Therefore, NPRWR is considered to perform better than the other models.

### 3.4. Isolated diseases and new miRNA prediction

**Fig 4:**
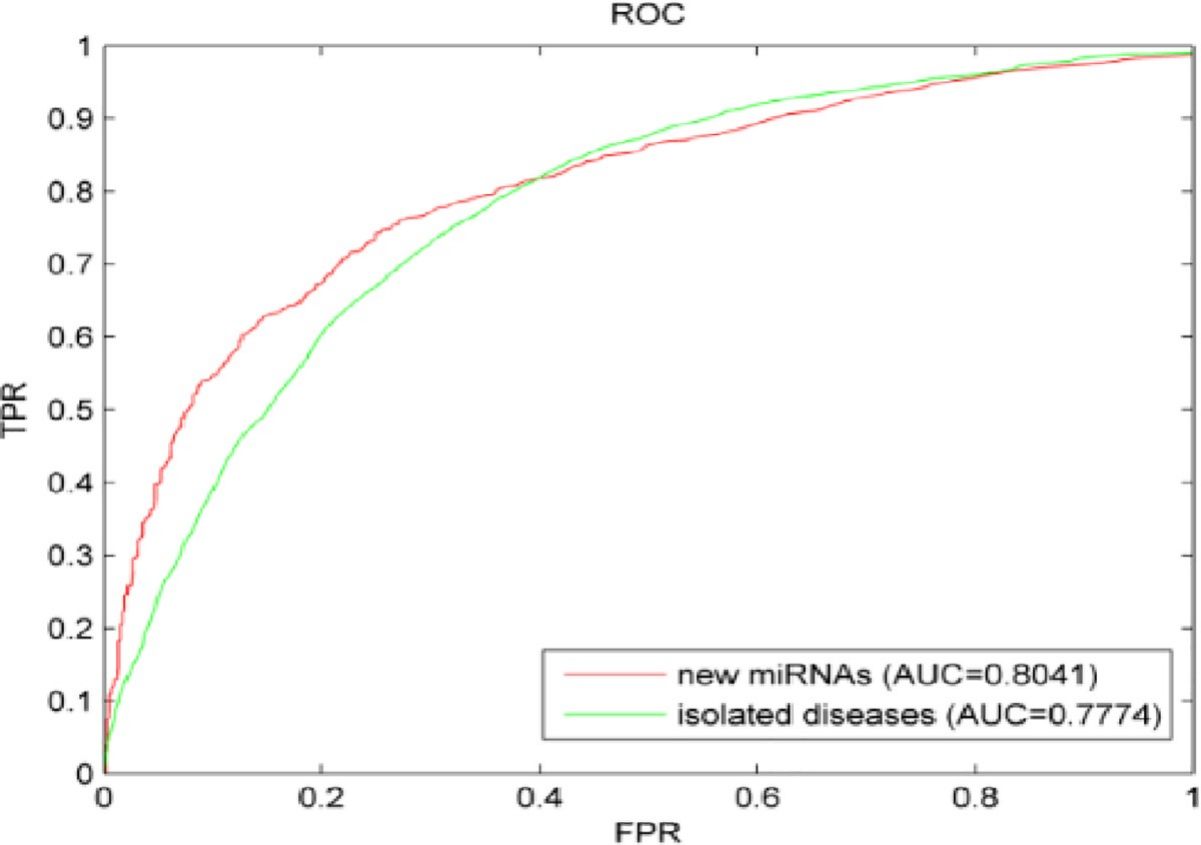
Predictions of new miRNAs and isolated diseases.

Isolated diseases refer to diseases in which the miRNA-associated information is completely unknown. The known association between the disease to be queried and all miRNAs was removed to simulate isolated diseases. In the cross verification, a disease was simulated as an isolated disease. Then, the remaining known information was used as basis to implement NPRWR for prediction. This step was repeated until each disease was predicted once as a test sample. The prediction result was evaluated by the ROC curve and AUC value. Fig 4 shows the prediction results. The AUC value was 0.7774, indicating that the method proposed here is effective in the prediction of isolated disease–miRNA relationship.

In recent years, more miRNAs have been discovered. However, their relation to diseases is mostly unknown, thus posing a great challenge to the prediction algorithm. The existing prediction methods cannot solve these problems. All predicted miRNA–disease association information should be removed to verify the effectiveness of the method proposed in this paper in the prediction of new miRNA–disease associations. NPRWR was implemented for prediction. As shown in Fig 4, the AUC value reached 0.8041 in the prediction of new miRNAs, indicating that our method has good performance in the prediction of new miRNA–disease associations.

### 3.5. Case study

Mutations and disorders of miRNA play an important role in the development of human diseases. The research on disease-related miRNAs aids in the diagnosis and treatment of diseases. Lung neoplasm and kidney neoplasm were selected to conduct a case analysis to further evaluate the prediction effect of NPRWR on potential miRNA–disease associations.

In the last 30 years, the number of newly discovered lung neoplasm has significantly increased. Early diagnosis of lung neoplasm is helpful for the treatment of tumors [72]. In our data, 132 miRNAs are associated with the occurrence and development of lung neoplasm. In this paper, NPRWR was adopted to perform lung neoplasm experiment based on these known data. Among the first 50 miRNAs associated with lung neoplasm predicted by our method, the supporting evidence can be found from the HMDD 3.0 and dbDEMC data sets for 49 miRNAs. The two data sets contained no evidence for hsa-mir-451b. However, Natarelli [73] discovered that hsa-miR-451b can inhibit the lung metastasis of osteosarcoma(see Table 1).

**Table 1:** The top 50 lung neoplasm–related miRNAs.

Rank	miRNA name	evidences	Rank	miRNA name	evidences
1	hsa-mir-1254	dbDEMC	26	hsa-mir-372	HMDD,dbDEMC
2	hsa-mir-3940	dbDEMC	27	hsa-mir-10a	HMDD,dbDEMC
3	hsa-mir-106b	dbDEMC	28	hsa-mir-449a	HMDD,dbDEMC
4	hsa-mir-16	HMDD,dbDEMC	29	hsa-mir-302c	dbDEMC
5	hsa-mir-708	dbDEMC	30	hsa-mir-151b	dbDEMC
6	hsa-mir-15b	dbDEMC	31	hsa-mir-328	HMDD,dbDEMC
7	hsa-mir-487a	dbDEMC	32	hsa-mir-195	HMDD,dbDEMC
8	hsa-mir-1258	HMDD,dbDEMC	33	hsa-mir-144	HMDD,dbDEMC
9	hsa-mir-204	dbDEMC	34	hsa-mir-302a	dbDEMC
10	hsa-mir-193b	dbDEMC	35	hsa-mir-141	HMDD,dbDEMC
11	hsa-mir-130a	HMDD,dbDEMC	36	hsa-mir-337	dbDEMC
12	hsa-mir-424	dbDEMC	37	hsa-mir-520c	dbDEMC
13	hsa-mir-373	HMDD,dbDEMC	38	hsa-mir-28	dbDEMC
14	hsa-mir-302b	dbDEMC	39	hsa-mir-485	HMDD,dbDEMC
15	hsa-mir-20b	dbDEMC	40	hsa-mir-374a	HMDD,dbDEMC
16	hsa-mir-378a	dbDEMC	41	hsa-mir-668	dbDEMC
17	hsa-mir-451b	[73]	42	hsa-mir-153	HMDD,dbDEMC
18	hsa-mir-15a	HMDD,dbDEMC	43	hsa-mir-23b	dbDEMC
19	hsa-mir-429	dbDEMC	44	hsa-mir-361	HMDD,dbDEMC
20	hsa-mir-451a	HMDD,dbDEMC	45	hsa-mir-345	dbDEMC
21	hsa-mir-92b	dbDEMC	46	hsa-mir-449b	dbDEMC
22	hsa-mir-625	dbDEMC	47	hsa-mir-520a	dbDEMC
23	hsa-mir-151a	dbDEMC	48	hsa-mir-302d	dbDEMC
24	hsa-mir-149	HMDD,dbDEMC	49	hsa-mir-152	HMDD,dbDEMC
25	hsa-mir-99a	HMDD,dbDEMC	50	hsa-mir-520b	HMDD,dbDEMC

For kidney neoplasm, among the first 50 miRNAs associated with lung neoplasm, supporting evidence can be found from the HMDD 3.0 and dbDEMC data sets for 49 miRNAs. No evidence can be found for hsa-mir-1(see Table 2).

**Table 2:** The top 50 kidney neoplasm–related miRNAs.

Rank	miRNA name	evidences	Rank	miRNA name	evidences
1	hsa-mir-155	HMDD,dbDEMC	26	hsa-mir-134	dbDEMC
2	hsa-mir-146a	dbDEMC	27	hsa-mir-7	dbDEMC
3	hsa-mir-122	HMDD,dbDEMC	28	hsa-mir-17	HMDD,dbDEMC
4	hsa-mir-34a	HMDD,dbDEMC	29	hsa-mir-142	dbDEMC
5	hsa-mir-221	dbDEMC	30	hsa-mir-708	HMDD
6	hsa-mir-125b	dbDEMC	31	hsa-mir-9	HMDD,dbDEMC
7	hsa-mir-16	dbDEMC	32	hsa-mir-184	dbDEMC
8	hsa-mir-29a	dbDEMC	33	hsa-mir-106b	dbDEMC
9	hsa-mir-210	HMDD,dbDEMC	34	hsa-mir-148a	dbDEMC
10	hsa-mir-31	dbDEMC	35	hsa-mir-19a	dbDEMC
11	hsa-mir-29b	dbDEMC	36	hsa-mir-27a	HMDD,dbDEMC
12	hsa-mir-199a	HMDD,dbDEMC	37	hsa-mir-1207	dbDEMC
13	hsa-mir-26a	dbDEMC	38	hsa-mir-19b	dbDEMC
14	hsa-mir-145	dbDEMC	39	hsa-mir-373	dbDEMC
15	hsa-mir-133a	dbDEMC	40	hsa-let-7b	dbDEMC
16	hsa-mir-222	dbDEMC	41	hsa-mir-200a	HMDD,dbDEMC
17	hsa-mir-196a	dbDEMC	42	hsa-mir-126	HMDD,dbDEMC
18	hsa-mir-206	dbDEMC	43	hsa-mir-137	dbDEMC
19	hsa-mir-20a	dbDEMC	44	hsa-mir-30b	dbDEMC
20	hsa-mir-1	Unconfirmed	45	hsa-mir-34c	dbDEMC
21	hsa-mir-200b	dbDEMC	46	hsa-mir-212	dbDEMC
22	hsa-mir-15b	dbDEMC	47	hsa-let-7a	dbDEMC
23	hsa-mir-218	dbDEMC	48	hsa-mir-92a	dbDEMC
24	hsa-mir-29c	dbDEMC	49	hsa-mir-124	dbDEMC
25	hsa-mir-223	dbDEMC	50	hsa-mir-204	dbDEMC

The known miRNAs associated with the diseases being verified were deleted to evaluate the performance of NPRWR in the prediction of isolated diseases. This operation can ensure that we only used the similarity information between the disease being verified and other diseases and the miRNA information associated with other diseases. For lung neoplasm, 132 known lung neoplasm–miRNA associations were deleted. NPRWR was used to predict the potential miRNA–lung neoplasm association. The first 50 miRNAs that were predicted can be found in HMDD and dbDEMC databases (see Table 3). For kidney neoplasm, seven known associations were deleted to make prediction by implementing NPRWR. In the prediction results, of the first 50 prediction associations, 48 had evidence stored in HMDD and dbDEMC databases. The two databases contained no evidence for hsa-mir-1 and hsa-mir-9(see Table 4). In the prediction of common diseases, hsa-mir-1 is associated with kidney neoplasm. In the future, scientists can find evidence for hsa-mir-1 and hsa-mir-9 association with kidney neoplasm.

**Table 3:** The top 50 lung neoplasms–related miRNAs candidates predicted by NPRWR with removed all known lung neoplasms–miRNAs associations and the confirmation of these associations.

Rank	miRNA name	evidences	Rank	miRNA name	evidences
1	has-mir-21	HMDD,dbDEMC	26	has-mir-106b	dbDEMC
2	has-mir-155	HMDD,dbDEMC	27	has-mir-7	HMDD,dbDEMC
3	has-mir-146a	HMDD,dbDEMC	28	has-mir-223	HMDD,dbDEMC
4	has-mir-122	HMDD,dbDEMC	29	has-mir-137	HMDD,dbDEMC
5	has-mir-34a	HMDD,dbDEMC	30	has-mir-218	HMDD,dbDEMC
6	has-mir-221	HMDD,dbDEMC	31	has-mir-148a	HMDD,dbDEMC
7	has-mir-16	HMDD,dbDEMC	32	has-mir-200b	HMDD,dbDEMC
8	has-mir-125b	HMDD,dbDEMC	33	has-mir-212	HMDD,dbDEMC
9	has-mir-31	HMDD,dbDEMC	34	has-mir-29c	HMDD,dbDEMC
10	has-mir-29a	HMDD,dbDEMC	35	has-mir-142	HMDD,dbDEMC
11	has-mir-26a	HMDD,dbDEMC	36	has-mir-17	HMDD,dbDEMC
12	has-mir-210	HMDD,dbDEMC	37	has-mir-27a	HMDD,dbDEMC
13	has-mir-133a	HMDD,dbDEMC	38	has-mir-373	HMDD,dbDEMC
14	has-mir-134	HMDD,dbDEMC	39	has-mir-34c	HMDD,dbDEMC
15	has-mir-29b	HMDD,dbDEMC	40	has-let-7b	HMDD,dbDEMC
16	has-mir-199a	HMDD,dbDEMC	41	has-mir-9	HMDD,dbDEMC
17	has-mir-222	HMDD,dbDEMC	42	has-mir-19a	HMDD,dbDEMC
18	has-mir-196a	HMDD,dbDEMC	43	has-mir-132	HMDD,dbDEMC
19	has-mir-206	HMDD,dbDEMC	44	has-mir-30b	HMDD,dbDEMC
20	has-mir-1	HMDD,dbDEMC	45	has-mir-19b	HMDD,dbDEMC
21	has-mir-145	HMDD,dbDEMC	46	has-mir-1207	dbDEMC
22	has-mir-15a	HMDD,dbDEMC	47	has-mir-34b	HMDD,dbDEMC
23	has-mir-20a	HMDD,dbDEMC	48	has-mir-451a	HMDD,dbDEMC
24	has-mir-15b	HMDD,dbDEMC	49	has-mir-124	HMDD,dbDEMC
25	has-mir-184	HMDD,dbDEMC	50	has-mir-93	HMDD,dbDEMC

**Table 4:** The top 50 kidney neoplasms–related miRNAs candidates predicted by NPRWR with removed all known kidney neoplasms–miRNAs associations and the confirmation of these associations.

Rank	miRNA name	evidences	Rank	miRNA name	evidences
1	hsa-mir-21	HMDD,dbDEMC	26	hsa-mir-184	dbDEMC
2	hsa-mir-155	HMDD,dbDEMC	27	hsa-mir-218	dbDEMC
3	hsa-mir-146a	dbDEMC	28	hsa-mir-7	dbDEMC
4	hsa-mir-122	dbDEMC	29	hsa-mir-200b	dbDEMC
5	hsa-mir-34a	HMDD,dbDEMC	30	hsa-mir-223	dbDEMC
6	hsa-mir-221	dbDEMC	31	hsa-mir-29c	dbDEMC
7	hsa-mir-125b	dbDEMC	32	hsa-mir-142	dbDEMC
8	hsa-mir-16	dbDEMC	33	hsa-mir-137	dbDEMC
9	hsa-mir-29a	dbDEMC	34	hsa-mir-148a	dbDEMC
10	hsa-mir-31	dbDEMC	35	hsa-mir-17	HMDD,dbDEMC
11	hsa-mir-210	HMDD,dbDEMC	36	hsa-mir-212	dbDEMC
12	hsa-mir-29b	dbDEMC	37	hsa-mir-106b	dbDEMC
13	hsa-mir-26a	HMDD,dbDEMC	38	hsa-mir-27a	HMDD,dbDEMC
14	hsa-mir-199a	HMDD,dbDEMC	39	hsa-mir-373	dbDEMC
15	hsa-mir-133a	dbDEMC	40	hsa-mir-708	dbDEMC
16	hsa-mir-196a	dbDEMC	41	hsa-mir-9	Unconfirmed
17	hsa-mir-222	dbDEMC	42	hsa-let-7b	dbDEMC
18	hsa-mir-206	dbDEMC	43	hsa-mir-19a	dbDEMC
19	hsa-mir-134	dbDEMC	44	hsa-mir-19b	dbDEMC
20	hsa-mir-145	dbDEMC	45	hsa-mir-132	dbDEMC
21	hsa-mir-1	Unconfirmed	46	hsa-mir-30b	dbDEMC
22	hsa-mir-20a	dbDEMC	47	hsa-mir-34c	dbDEMC
23	hsa-mir-15a	HMDD,dbDEMC	48	hsa-mir-451a	dbDEMC
24	hsa-mir-15b	dbDEMC	49	hsa-mir-124	dbDEMC
25	hsa-mir-1207	dbDEMC	50	hsa-mir-93	HMDD,dbDEMC

## 4. Discussion

In this paper, a NPRWR model based on dual random walk with restart and network projection was proposed to predict potential miRNA–disease associations. NPRWR not only exhibits high performance in the prediction of unknown miRNA–disease interactions but can also effectively predict isolated diseases and new miRNA.

To fairly evaluate the performance of the NPRWR model, we compared NPRWR with the most advanced models (MDHGI, NSEMDA, RFMDA, and SNMFMDA). The prediction scores of NPRWR, MDHGI, NSEMDA, RFMDA, and SNMFMDA were 0.9029, 0.8945, 0.8899, 0.8891, and 0.9007, respectively. NPRWR yielded the best prediction results compared with the other methods.

Each disease (miRNA) was simulated as an isolated disease (new miRNA) to evaluate the performance of NPRWR in the prediction of isolated diseases and new miRNAs. Then, cross verification was carried out for each disease (miRNA). The AUC values were 0.7774 and 0.8041, indicating that our method has good prediction effect on the prediction of relationships between isolated diseases and miRNA.

In addition, lung neoplasm and kidney neoplasm were selected to conduct a case analysis to further verify the reliability of the NPRWR model in the prediction of potential relationships between miRNA and diseases. In the prediction of common diseases, of the first 50 miRNAs obtained in the prediction of the two diseases, 49 had evidence stored in HMDD or dbDEMC databases. For the prediction of isolated diseases, in the first 50 miRNAs associated with lung neoplasm obtained by NPRWR prediction, supporting evidence can be found from known databases. For the 48 of the first 50 miRNAs associated with kidney neoplasm, supporting evidence can be found from HMDD or dbDEMC databases. No evidence can be found for hsa-mir-1 and hsa-mir-9.

In conclusion, NPRWR is simple to use and can be applied to the prediction of isolated diseases and new miRNAs, showing strong interpretability and requiring several parameters. The model can also be used to make prediction by using limited resources. Therefore, the calculation method we proposed can be used as a powerful auxiliary tool for biological experiments. However, NPRWR has defects. First, the construction of disease similarity network and miRNA similarity network lacks scientificity. The accuracy of common neighbor link prediction algorithm based on disease functional similarity declines. Second, in consideration that the associations between available miRNAs verified experimentally and diseases are still relatively limited, and miRNA similarity is calculated based on such associations, NPRWR may generate biased predictions.
